# Validating abortion procedure coding in Canadian administrative databases

**DOI:** 10.1186/s12913-016-1485-4

**Published:** 2016-07-12

**Authors:** Saied Samiedaluie, Sandra Peterson, Rollin Brant, Janusz Kaczorowski, Wendy V. Norman

**Affiliations:** Department of Family Practice, University of British Columbia, Vancouver, Canada; Centre for Health Services and Policy Research, School of Population and Public Health, University of British Columbia, Vancouver, Canada; Department of Statistics, University of British Columbia, Vancouver, Canada; Département de médecine de famille et de médecine d’urgence, l’Université de Montréal, Montréal, Canada

**Keywords:** Induced abortion, Clinical coding, Database, Data collection, Reproducibility of results, British Columbia

## Abstract

**Background:**

The British Columbia (BC) Ministry of Health collects abortion procedure data in the Medical Services Plan (MSP) physician billings database and in the hospital information Discharge Abstracts Database (DAD). Our study seeks to validate abortion procedure coding in these databases.

**Methods:**

Two randomized controlled trials enrolled a cohort of 1031 women undergoing abortion. The researcher collected database includes both enrollment and follow up chart review data. The study cohort was linked to MSP and DAD data to identify all abortions events captured in the administrative databases. We compared clinical chart data on abortion procedures with health administrative data. We considered a match to occur if an abortion related code was found in administrative data within 30 days of the date of the same event documented in a clinical chart.

**Results:**

Among 1158 abortion events performed during enrollment and follow-up period, 99.1 % were found in at least one of the administrative data sources. The sensitivities for the two databases, evaluated using a gold standard, were 97.7 % (95 % confidence interval (CI): 96.6–98.5) for the MSP database and 91.9 % (95 % CI: 90.0–93.4) for the DAD.

**Conclusions:**

Abortion events coded in the BC health administrative databases are highly accurate. Single-payer health administrative databases at the provincial level in Canada have the potential to offer valid data reflecting abortion events.

**Trial registration:**

ClinicalTrials.gov Identifier NCT01174225, Current Controlled Trials ISRCTN19506752.

## Background

Accurate capture of abortion events within administrative data is important for both population health surveillance and for policy and program planning and evaluation. Abortion is a common procedure in Canada, with 92,524 reported in 2011 including 14,341 in the province of British Columbia (BC), and 37 % of women seeking a hospital-provided abortion in 2011 reporting having had at least one prior abortion [1]. Interventions that assist women presenting for an abortion to avoid subsequent unintended pregnancies have the potential to address this problem, yet clinical follow up post-abortion has a very high attrition rate. Health administrative data could potentially be used to test the effectiveness of health policies and programs with potential to impact the number of abortions performed. However, we were unable to find any evidence that the validity of the data capture of these events within a single-payer health administrative data system in Canada has ever been documented.

Observation of outcomes after an index abortion presents unique challenges, due to very low rates of return for clinical follow up post abortion [2, 3]. Failure to return for follow up is frequently associated with lower socioeconomic status, of particular concern as women of low socioeconomic status are over-represented among the population presenting for abortion, and particularly for repeat abortion [2, 4, 5]. Comprehensive chart review among all clinical services offering abortion within a jurisdiction, while theoretically possible, is time-consuming, costly and impeded by the wide range of medical record systems used. Further, chart audit methodology is likely to decline in accuracy due to the recent approval in Canada of mifepristone (RU-486) for use to induce medical abortion, which may be performed in a wide range of primary care settings otherwise unrelated to settings self-identifying as abortion clinics [6].

Capture of abortion events using health administrative data has the potential to provide comprehensive data on both numbers and rates of abortion in relation to a wide range of determinants, as well as in relation to subsequent health system events. Administrative data capture could be associated with lower costs than chart review and with greater accuracy for subsequent events than clinical follow up. Administrative databases have the added advantage of allowing for extraction of additional information, such as delivery outcomes and hospitalization records, which could be of value to inform health policy and system decision making.

The accuracy of using health administrative data to capture abortion events is currently unknown as no validation of abortion procedure capture and coding in administrative data compared to verified clinical records has been undertaken. Previous Canadian studies on administrative data capture have shown the validity of administrative coding for cardiac and perinatal procedures [7–9]. Based on these results, we anticipate a high degree of accuracy in administrative coding for abortions.

We conducted two randomized controlled trials (RCTs) enrolling over a thousand women presenting for abortion in BC [5, 10–12]. We followed up each participant through the review of clinical chart records from all six abortion clinic facilities in BC for both index and subsequent abortions. We examined the analogous data for each participant as captured through the provincial health administrative linked databases of Population Data BC, and the BC Ministry of Health [13–16]. By comparing these different data sources, we evaluated the validity of using health administrative data to capture abortion events among residents of BC registered in the provincial health system.

## Methods

### Study data

From 2009 to 2012, two RCTs studying the effectiveness of intrauterine contraceptive devices enrolled a cohort of 1031 women presenting for surgical abortion at BC clinics [5, 10–12]. Women were screened using the eligibility criteria of the studies. Two main conditions for being eligible to participate were the intention not to conceive within the subsequent year and current registration with the BC provincial health plan (Medical Services Plan). Over three years, the two studies recruited 530 women undergoing a first trimester abortion and 501 women undergoing a second trimester abortion. Studies were carried out in five BC surgical abortion clinics, of which two are hospital-based and three are in the community setting. Chart review was conducted at these five clinics, as well as at an additional community based clinic which offers only medical abortion, to capture subsequent events among enrolled participants.

Women enrolling in the study gave consent for follow up clinically and through linkages using health administrative data, as well as through the completion of annual questionnaires. The participants were followed subsequently for five years including by an annual chart review at all six abortion clinics in BC. The chart review data set, including initial enrollment data as well as direct confirmation of individual initial and subsequent clinical events whenever it was required, constitutes the researcher-collected database for this study.

The trials were conducted under the supervision of an independent Data and Safety Monitoring Board. Ethics approval for the trials, including the current secondary analysis, was obtained from the University of British Columbia- Children’s and Women’s Hospital Research Ethics Board (REB) (H10-00306, H10-00798) and from the analogous REBs for all health authorities with study sites. Both study protocols, and the intake cohort characteristics for one RCT, have been published [5, 10–12].

### Administrative data

In BC, administrative data relevant to abortion care are captured through diagnostic and procedural coding for fees billed by physicians in the Medical Services Plan (MSP) Payment Information database, and diagnostic and procedural coding at hospital discharge in the Discharge Abstracts Database (DAD). There are several research conducted based on the data captures in these databases [17–22]. Different characteristics of MSP and DAD databases are described in Table 1. Abortions are identified in the MSP data with ICD-9 codes 635.x-638.x, and fee item codes 4110-4114, 14545, and, if performed in known abortion facilities by physicians consistently coded as providing abortion services, the induction of abortion is indicated by the code 0787 [23]. In the hospital DAD data abortions are identified with ICD-10-CA code O04 and CCI procedure codes beginning with 5CA. We analyzed MSP coding for fee items related to abortion procedures (i.e., 17B: consult for abortion) to investigate procedures that were missing codes or were in unexpected date ranges.

**Table 1:** Characteristics of administrative database capturing abortions data in BC

Characteristic	Medical Services Plan (MSP) Payment Information	Discharge Abstract Database
Coverage	Individuals covered by the Medical Services Plan (MSP), BC’s universal insurance program	In-patients and day surgery patients in acute care hospitals in BC
Data	Billing information for all medically required services (both procedure and diagnostic codes)	Data on discharges, transfers and deaths
Provided by	Fee-for-service practitioners	Acute care hospitals
Abortion codes	ICD-9 codes 635.x-638.x	ICD-10-CA code O04
	fee item codes 4110-4114, 14545	CCI procedure codes beginning with 5CA.
	code 0787	

The study cohort was linked to the administrative data in 2014 using personal health numbers (PHN) and date of birth as unique patient identifiers. Administrative data for all index and subsequent abortion procedures occurring for the study cohort between June 1, 2009 and September 30, 2013 were captured. We required the women’s consent during enrollment to perform this data linkage as the abortion data in these two health databases is not publicly available. Using hospital and anonymous physician codes, we excluded, for the purposes of this comparison, any procedures that did not occur at the six clinics for which we had access to clinical charts.

### Analysis

There are two sets of abortions events in our study that we can use to validate the procedure coding of abortions; the index abortions, occurring at the time of enrollment in either of the two RCTs; and the subsequent abortions, which occurred within the 5-year follow up timeline. All discrepancies between data sources were investigated through chart review at the relevant facility.

When comparing the administrative data with the researcher-collected medical chart data, we considered an abortion event to be matched if an abortion procedure code exists in the relevant administrative data source with a date that is accurate within ±30 days compared to the researcher-collected data. In cases with multiple abortion related procedural codes within a window of 30 days, we assumed it to be a single abortion event, an assumption that was universally supported by review of chart data. A common observation in the MSP data was the presence of abortion-related diagnostic codes (such as at the time of provision of consultation, follow up, or complications) in the absence of an abortion procedure code. We examined these cases but once all inconsistencies were resolved through specific clinical chart confirmation, we were able to restrict our consideration to cases with an abortion procedural code for the purpose of matching between databases.

We report discrepancies and the source, if known, of errors or omissions in the administrative data. In the analysis of index abortions, sensitivity is defined as the ratio of correct matches of the administrative data with the researcher-collected medical chart data. Since the abortions are recorded in two administrative databases in BC, we provide the sensitivity statistics for each data source separately as well as for the two databases combined.

In the analysis of subsequent abortions, we found abortions performed at study related facilities identified in administrative data that were not present in researcher-collected data. Therefore, for this cohort of abortions we conduct a two-direction analysis to measure the number of matched events in the administrative and researcher-collected databases. When reporting the results of our comparisons, we also include the exact binomial confidence intervals calculated using the Clopper-Pearson method. All the statistical analysis in this paper was conducted using the statistical software R.

## Results

We present the results of our analysis for index abortion events separately from our consideration of subsequent abortion events.

### Index abortions

**Fig. 1:**
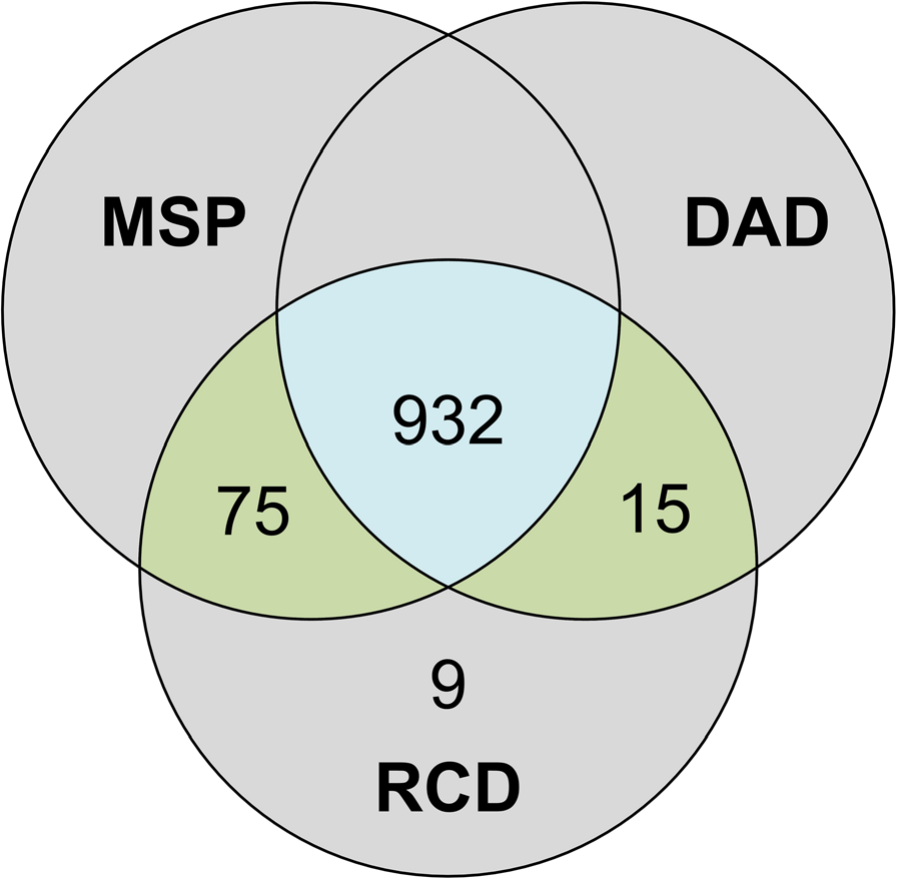
Number of the index abortions found in different data sources. Legend: RCD = Research Collected Database, MSP = Medical Service Plan Payment Database, DAD = Discharge Abstract Database

There were 1031 women enrolled in our studies. Out of the 1031 index abortions that were registered in our researcher-collected database, 1022 events (99.1 %, 95 % confidence interval (CI): 98.3–99.6) were matched in at least one of the two administrative data sources, and 932 (90.4 %, 95 % CI: 88.4–92.1) were found in both. There were nine cases that did not match to any MSP or DAD data (all cases conducted at a non-hospital setting). Overall, the MSP data correctly captured 1007 of 1031 events (97.7 %) and the DAD captured 947 (91.9 %). Among the 24 abortion events that were not found in the MSP, abortion-related codes (such as consultation or follow up) were present for 13 of them. Moreover, we found two cases where MSP procedure codes had been submitted twice for the same procedure. Figure 1 shows the number of index abortions in the researcher-collected database matched in the administrative datasets.

Table 2 shows the sensitivity of the two administrative data sources and the combination of the two (events that were correctly matched in at least one of the two databases) for the index abortions. The comparative sensitivities are 97.7 % (95 % CI: 96.6–98.5) for the MSP data and 91.9 % (95 % CI: 90.0–93.4) for the DAD. The difference in sensitivity between MSP and DAD data is statistically significant for the index abortions. When the two administrative data sources are used together for identifying the index abortion procedures, the relative sensitivity to the gold standard (researcher-collected data) is 99.1 % (95 % CI: 98.3–99.6).

**Table 2:** Sensitivity of administrative databases for the index abortions when compared to the researcher-collected database

Source	Sensitivity (95 % CI)
MSP or DAD	99.1 (98.3–99.6)
MSP	97.7 (96.6–98.5)
DAD	91.9 (90.0–93.4)

*MSP* Medical Service Plan Payment Database, *DAD* Discharge Abstracts Database

### Subsequent abortions

There were 125 subsequent abortions found in the researcher-collected data within the time-frame of this analysis, out of which 124 events were matched in at least one of the administrative data sources. However, over the same time period, there were two abortions that were identified in the administrative databases but were not captured by the researcher collected medical chart review database. Therefore, a total of 127 abortion events have occurred which are confirmed by at least one of the available data sources. There were also two abortion procedures found in the DAD that did not reflect actual abortion procedures, as confirmed through review of the clinical charts. Table 3 shows the number of subsequent abortions found in each of the administrative data and research-collected data sources and the number of events that were matched by the other database.

**Table 3:** Number of subsequent abortions events found in different data sources

Source	N	Proportion (95 % CI)
Total subsequent abortions found in all databases	127	Reference
Matched in RCD	125	98.4 (94.4–99.8)
Matched in MSP or DAD	126	99.2 (95.7–100)
MSP	126	99.2 (95.7–100)
DAD	78	61.4 (52.4–69.9)
Matched in both admin and RCD	124	97.6 (93.3–99.5)

*RCD* Research Collected Database, *MSP* Medical Service Plan Payment Database, *DAD* Discharge Abstracts Database

### Event date difference

Of all index and subsequent abortions matched within our 30 day window (1146 events), 99.1 % (95 % CI: 98.4–99.6) had the same date in researcher-collected and administrative data sources and 99.6 % (95 % CI: 99.0–99.9) were discrepant by one day or less. Table 4 provides additional detail of this date comparison. The majority of the date discrepancies occurred in cases where the abortion procedure was conducted over multiple days.

**Table 4:** Event date difference among all data sources for index and subsequent abortions

Days difference	Frequency	Percent	Cumulative Frequency	Cumulative Percent
−30	1	0.09	1	0.09
−7	1	0.09	2	0.17
−3	1	0.09	3	0.26
−1	2	0.17	5	0.44
0	1136	99.13	1141	99.56
1	3	0.26	1144	99.83
2	1	0.09	1145	99.91
6	1	0.09	1146	100

## Discussion

This is the first study to examine the validity of administrative data capture of abortion procedure events in Canada. We found a high degree of accuracy, with over 99 % of procedures being correctly identified by at least one of the two administrative databases.

While the two administrative data sources combined had a high sensitivity (99.1 %), there was a significant difference in the concordance rates between the MSP and DAD for the index and subsequent abortion events. The accuracy of the hospital discharge data was lower compared to fee for service data (MSP), 91.9 % versus 97.7 % sensitivity among the index cases. The match rate for DAD was even less in the set of subsequent abortions, where only 61.4 % of cases were matched. The large difference in capturing the index and subsequent abortion events was expected. The majority of study participants had their index abortion in a hospital setting but only for these was the procedure captured in the hospital discharge data. Community setting clinics, where some of the index abortions and a large portion of the subsequent abortions occurred do not contribute to the hospital discharge data. Across Canada more than half of all abortions reporting location of service delivery are performed in clinic settings [1]. Thus, the hospital discharge abstract database (DAD) is not reliable as an independent source for capturing abortions in Canada.

The high capture rate of abortions events by the combined BC administrative databases suggests that this technique can be used as a reliable source in abortion related studies in BC. Based on the access, cost and time needed to collect data from available sources, researcher-collected compared to administrative databases, the prospective data user can decide the most appropriate technique. Researcher-collected clinical chart data is likely to require individual consent as well as, or at least with, ethics review board approval. The variety of clinical data storage systems (paper and a number of different electronic medical records) may make collection of clinic-based data cumbersome and expensive. However, the clinical chart data is available immediately after the event, while data capture in administrative data sources may require 18–24 months prior to availability. Further, permission to access the data may require additional time [24]. Thus the potential data user must weigh the various practical considerations to choose an appropriate data source, as our results do not present a significant variation in the accuracy of the data available from either source.

We were limited in this study by our inability to capture chart data from all abortion settings in BC. In 2010, less than 10 % of abortions in BC occurred in clinics not involved in this study [25]. Among the study cohort, administrative data found only 1.7 % of women presented for a subsequent abortion at a location for which we did not have corresponding clinical chart data. Thus this limitation did not significantly affect our ability to validate the capture of subsequent abortion outcomes using these administrative data. Similarly, another limitation is the exclusion of non-BC residents among the index abortion sample. This exclusion (which was necessary as MSP information does not exist for non-residents) accounted for only 3.5 % of patients assessed for eligibility. We feel that these limitations impact neither the validity of administrative data use in the context of clinical trials, nor the capture and reporting of abortion data at the provincial level.

This analysis has focused on the validation of the capture of clinical abortion events in the BC government health administrative databases. The results may have limited application to the capture of abortion events in the administrative data of other jurisdictions. The Canadian Institute for Health Information (CIHI) has found that the use of fee for service data, analogous to MSP data in BC, to record abortions varies significantly between Canadian provinces [1]. This accords with our findings where the combination of MSP and hospital discharge data provided a better capture of events. The ability to combine linked administrative data from both of these sources, and to compare to clinical chart recorded events, is a major strength of this study.

## Conclusions

Abortion procedures are common and of interest to health researchers, health policy makers and health program planners. The capture of abortion procedure events in health administrative data in Canada has not been previously validated. The high degree of accurate capture (over 99 %) that we found validates the use of linked BC health administrative data to capture abortion procedure events.

## Abbreviations

BC, British Columbia; CI, confidence interval; CIHI, Canadian Institute for Health Information; DAD, Discharge Abstracts Database (representing hospital services and diagnostic data in BC); MSP, Medical Services Plan (representing physician services and diagnostic billing data in BC); PHN, personal health number; RCD, Research Collected Database; RCT, randomized controlled trial; REB, Research Ethics Board; WHRI, Women’s Health Research Institute

## References

[1] Canadian Institute for Health Information (2014). Induced abortions performed in Canada in 2011.

[2] Stanek AM, Bednarek PH, Nichols MD, Jensen JT, Edelman AB (2009). Barriers associated with the failure to return for intrauterine device insertion following first-trimester abortion. Contraception.

[3] Norman WV (2012). Induced abortion in Canada 1974–2005: trends over the first generation with legal access. Contraception.

[4] Bednarek PH, Creinin MD, Reeves MF, Cwiak C, Espey E, Jensen JT (2011). Immediate versus delayed IUD insertion after uterine aspiration. N Engl J Med.

[5] Norman WV, Chiles J, Turner C. The contraceptive experience among women seeking abortion. Contraception. 2011;84(3):314.

[6] Health Canada (2015). Regulatory decision summary (SBD): MIFEGYMISO - 2015 - Health Canada.

[7] Lee DS, Stitt A, Wang X, Yu JS, Gurevich Y, Kingsbury KJ, Austin PC, Tu JV (2013). Administrative hospitalization database validation of cardiac procedure codes. Med Care.

[8] Joseph KS, Fahey J (2009). Validation of perinatal data in the discharge abstract database of the Canadian Institute for Health Information. Chronic Dis Can.

[9] Frosst G, Hutcheon J, Joseph KS, Kinniburgh B, Johnson C, Lee L (2015). Validating the British Columbia perinatal data registry: a chart re-abstraction study. BMC Pregnancy Childbirth.

[10] Norman WV, Kaczorowski J, Soon JA, Brant R, Bryan S, Trouton KJ, Dicus L (2011). Immediate vs. delayed insertion of intrauterine contraception after second trimester abortion: study protocol for a randomized controlled trial. Trials.

[11] Norman WV, Chiles JL, Turner CA, Brant R, Aslan A, Kaczorowski J (2012). Comparing the effectiveness of copper intrauterine devices available in Canada. Is FlexiT non-inferior to NovaT when inserted immediately after first-trimester abortion? study protocol for a randomized controlled trial. Trials.

[12] Norman WV, Brooks M, Brant R, Soon JA, Majdzadeh A, Kaczorowski J (2014). What proportion of Canadian women will accept an intrauterine contraceptive at the time of second trimester abortion? baseline data from a randomized controlled trial. J Obstet Gynaecol Can.

[13] British Columbia Ministry of Health. Medical Services Plan (MSP) Payment Information File. Population Data BC. Data Extract. MOH (2014). 2014. http://www.popdata.bc.ca/data.

[14] British Columbia Ministry of Health. Consolidation File (MSP Registration & Premium Billing). Population Data BC. Data Extract. MOH (2014). 2015. http://www.popdata.bc.ca/data

[15] British Columbia Vital Statistics Agency. Vital Statistics Deaths. Population Data BC. Data Extract. BC Vital Statistics Agency (2014). 2014. http://www.popdata.bc.ca/data.

[16] Canadian Institute for Health Information. Discharge Abstract Database (Hospital Separations). Population Data BC. Data Extract. MOH (2014). 2014. http://www.popdata.bc.ca/data.

[17] Lisonkova S, Liu S, Bartholomew S, Liston RM, Joseph KS (2011). Temporal trends in maternal mortality in Canada II: estimates based on hospitalization data. J Obstet Gynaecol Can.

[18] Smolina K, Hanley GE, Mintzes B, Oberlander TF, Morgan S (2015). Trends and determinants of prescription drug use during pregnancy and postpartum in British Columbia, 2002–2011: a population-based cohort study. PLoS One.

[19] Leung VW, Soon JA, Lynd LD, Marra CA, Levine M (2016). Population-based evaluation of the effectiveness of two regimens for emergency contraception. Int J Gynecol Obstet.

[20] Bedouch P, Marra CA, FitzGerald JM, Lynd LD, Sadatsafavi M (2012). Trends in asthma-related direct medical costs from 2002 to 2007 in British Columbia, Canada: a population based-cohort study. PLoS One.

[21] Chamberlayne R, Green B, Barer ML, Hertzman C (1998). Creating a population-based linked health database: a new resource for health services research. Can J Public Health.

[22] Liu S, Liston RM, Joseph KS, Heaman M, Sauve R, Kramer MS, Maternal Health Study Group of the Canadian Perinatal Surveillance System (2007). Maternal mortality and severe morbidity associated with low-risk planned cesarean delivery versus planned vaginal delivery at term. Can Med Assoc J.

[23] Doctors of BC (2015). Doctors of BC Guide to Fees.

[24] Population Data BC (2014). The data access request (DAR) process.

[25] Norman WV, Soon JA, Maughn N, Dressler J (2013). Barriers to rural induced abortion services in Canada: findings of the British Columbia abortion providers survey (BCAPS). PLoS One.

